# Identification of Anti-Proliferative Compounds from *Genista monspessulana* Seeds through Covariate-Based Integration of Chemical Fingerprints and Bioactivity Datasets

**DOI:** 10.3390/molecules27133996

**Published:** 2022-06-22

**Authors:** Luis Díaz, Willy Cely-Veloza, Ericsson Coy-Barrera

**Affiliations:** 1 Bioprospecting Research Group, School of Engineering, Universidad de La Sabana, Chía 250001, Colombia; 2 Bioorganic Chemistry Laboratory, Universidad Militar Nueva Granada, Cajicá 250247, Colombia; u7700102@unimilitar.edu.co

**Keywords:** Fabaceae, *Genista monspessulana*, metabolic profiling, alkaloids, isoflavones

## Abstract

*Genista monspessulana* (L.) L.A.S. Johnson (Fabaceae) is a Mediterranean plant introduced to South America and other regions for ornamental purposes. However, it is considered an invasive shrub due to its reproductive vigor in many areas. Unlike other *Genista* plants, *G. monspessulana* has few studies disclosing its biologically active components, particularly cytotoxic agents against cancer cells. Thus, as part of our research on anti-proliferative bioactives, a set of ethanolic seed extracts from ten accessions of *G. monspessulana*, collected in the Bogotá plateau, were evaluated against four cell lines: PC-3 (prostate adenocarcinoma), SiHa (cervical carcinoma), A549 (lung carcinoma), and L929 (normal mouse fibroblasts). Extracts were also analyzed through liquid chromatography coupled with mass spectrometry (LC/MS) to record chemical fingerprints and determine the composition and metabolite variability between accessions. Using multiple covariate statistics, chemical and bioactivity datasets were integrated to recognize patterns and identify bioactive compounds among studied extracts. *G. monspessulana* seed-derived extracts exhibited dose-dependent antiproliferative activity on PC-3 and SiHa cell lines (>500 µg/mL < IC_50_ < 26.3 µg/mL). Seven compounds (**1**–**7**) were inferred as the compounds most likely responsible for the observed anti-proliferative activity and subsequently isolated and identified by spectroscopic techniques. A tricyclic quinolizidine (**1**) and a pyranoisoflavone (**2**) were found to be the most active compounds, exhibiting selectivity against PC-3 cell lines (IC_50_ < 18.6 µM). These compounds were used as precursors to obtain a quinolizidine-pyranoisoflavone adduct via Betti reaction, improving the activity against PC-3 and comparable to curcumin as the positive control. Results indicated that this composition–activity associative approach is advantageous to finding those bioactive principles efficiently within active extracts. This correlative association can be employed in further studies focused on the targeted isolation of anti-proliferative compounds from *Genista* plants and accessions.

## 1. Introduction

*Genista* is a plant genus within the Fabaceae family that comprises brooms and gathers ca. 90 perennial shrubby and short woody species, which are generally accepted to have Mediterranean origin [1], and are usually employed as ornamentals due to their abundant blooms and often sweet smell [2]. An interesting plant within this genus is *Genista monspessulana* (L.) L.A.S. Johnson [=*Cytisus monspessulana* L.; *Teline monspessulana* (L.) K. Koch]. It is a perennial, upright honey shrub that can reach up to 3 m [3] and is native to the Mediterranean region, Canary Islands, north Africa, and western Asia. However, due to its flowering being predominantly continuous in tropical regions [4], this plant has been naturalized in Australia and North and South America [5,6]. In Colombia, *G. monspessulana* is well known by common names such as ‘smooth broom’ (i.e., ‘*retamilla*’ or ‘*escobilla*’) and French or cape broom [7,8]. It was introduced to Colombia as an ornamental plant, as living fences, and for controlling erosion on steep slopes and embankments, since it can form seed banks, holding vigor through its massive roots [9]. It also has high regeneration capacity, relevant regrowth ability with dense scrub associations, and adaptability to a broad range of soil types, particularly siliceous and acid soils [10]. Due to these features, this plant can also cause considerable damage and alterations to ecosystems into which it is introduced and, consequently, it is classified as an invasive plant [8,11]. Various strategies have been pursued to handle and control *G. monspessulana* invasion, and therefore, several studies have been developed to study its ecological relationships [12,13] and even its biomass exploitation [7,14,15].

*Genista* plants are well known for producing and accumulating flavonoids and alkaloids, which are the most representative phytoconstituents, and considered chemotaxonomic markers of the genus [16]. In addition, *Genista*-derived compounds and extracts have demonstrated several biological activities, such as estrogenic [17], anti-inflammatory [18], anti-diabetic [19], antioxidant [20], cytotoxic [21,22] and antiproliferative [23] activities. In this regard, anti-cancer-related actions represent an important biological activity of *Genista* plants [24]. In fact, they are a relevant source of phytoestrogenic isoflavones (e.g., daidzein and genistein and their derivatives), which may reduce the incidence of breast and prostate cancer [25]. However, there are few studies on the phytochemistry and biological activity of *G. monspessulana,* comprising merely the quantitative analysis and isolation of flavonoids and isoflavonoids [7,26,27], alkaloid profiling [28,29], and antioxidant activity evaluation [26]. Hence, this plant suffers from a lack of phytochemical and bioactivity studies; therefore, expanding knowledge on its chemical and biological characteristics, focused on the effects on cancer cell lines, constitutes the novelty of the present study.

Cancer is a health problem characterized by being one of the diseases with the highest mortality rate worldwide. According to the Global Cancer Observatory, in 2020, there were 10 million deaths from this disease around the world and 19.3 million new cases in the same year [30]. In Colombia, the numbers of new cases and deaths in 2020 were 113,221 and 54,987, respectively, with stomach (11.7%), lung (11.1%), breast (8.0%), colon (7.4%), prostate (7.0%), and cervix uteri (4.5%) being the cancer types with the highest mortality, although breast and prostate cancers had the highest incidence (13.7 and 12.8%, respectively) [30,31,32]. The drugs currently used for its treatment have limited activity and cause various undesirable effects, including cardiac, pulmonary, neurological, and renal toxicity [33]. Therefore, discovering new anticancer agents with higher efficacy and high selectivity for tumor cells is essential for reducing the number of deaths and even the side effects during and after chemotherapy [34]. In this context, plants are widely recognized as a key source of anticancer compounds, and *Genista* species have some representatives [24]. For instance, *G. sessilifolia* exhibited inhibitory activity against human histiocytic lymphoma (U937) and human melanoma (M14) cell lines [21,35], *G. ferox* against human cervical adenocarcinoma (HeLa) cell line [36], and *G. ulicina* against human colon cancer (HT-29) cell line [37], particularly involving (iso)flavonoids, alkaloids and saponins. Thus, *Genista* plants, e.g., *G. monspessulana*, can be examined to find compounds with anticancer properties.

Bioactive findings from natural sources can be accomplished through various approaches [38]. Small or large libraries comprising previously isolated compounds assessed through a standardized bioassay (conventional or high-throughput screening) or the consecutive evaluation of continuing-depurated fractions from an active parent extract (bioguided fractionation) are the most frequently used strategies to search for bioactives. However, they require time-consuming, laborious, and expensive protocols [39,40]. A recently emerging workflow, namely biochemometrics, can overcome these drawbacks by associating chemical and bioactivity datasets for discriminating activity-relating metabolites within plant fractions or extracts using multivariate statistics [41,42]. Thus, extracts obtained from several accessions can provide diversified chemical profiles and, consequently, statistical association with bioactivity to localize active components [43]. It is particularly favorable since specialized metabolism can be differentially regulated by biotic/abiotic factors and promote different bioactive and chemically diversified plant mixtures to be used as input for statistical analysis [44,45]. Combining chemical and bioactivity data from extracts of different accessions into a single examination is the main advantage of this integrative strategy, so the active principles highlighted by this covariate-based strategy can be subsequently isolated [46,47]. Therefore, it constitutes a beneficial starting point for focusing on the bioactive finding from plant sources [48].

Hence, as part of our research on recognizing naturally occurring compounds with anticancer properties, ethanol extracts from the seeds of ten *G. monspessulana* accessions were evaluated by the MTT (3-(4,5-dimethylthiazol-2-yl)-2,5-diphenyltetrazolium bromide) cell viability assay against three human cancer cell lines, i.e., lung carcinoma, cervical carcinoma, and prostatic adenocarcinoma, which are related to those cancer types with high mortality and incidence in Colombia [31]. The resulting anti-proliferative activity was statistically associated with mass spectrometry-based chemical composition, and seven compounds were recognized and identified using this integration.

## 2. Results and Discussion

*G. monspessulana* is widely distributed along the Bogotá plateau, particularly in open areas with higher light incidence [15]. These facts allow this plant to have rapid leaf and root development and microorganism association, facilitating nitrogen fixation [49]. Based on these facts, seeds from ten *G. monspessulana* accessions were retrieved from environmentally different locations to ensure a plausible chemical variation depending on growth conditions provided by surroundings [50]. Thus, the collected seeds per accession were subdivided into two accession groups (AG). AG1 (*Gm*1,2,4,6,8) comprised seeds of five accessions from open spaces and grasslands forming dense scrub associations and AG2 (*Gm*3,5,7,9,10) gathered those seeds of the other five accessions from spaces near woodland and even under a tree canopy. Ground, dry seeds were then extracted using 96% ethanol to afford crude extracts and subsequently used in the MTT cell viability assay. 

### 2.1. Anti-Proliferative Activity of G. monspessulana Seed-Derived Extracts 

*G. monspessulana* has no previous studies on bioactivity against cancer cell lines, but other *Genista* plants have shown cytotoxic/anti-proliferative activity due to alkaloids and flavonoids [24]. The anti-proliferative activity against three cancer cell lines (i.e., PC-3 (human prostate adenocarcinoma), SiHa (human cervical carcinoma), A549 (human lung carcinoma)), and a normal cell line (i.e., L929 (normal mouse fibroblasts)) of the ethanolic seed extracts (*n* = 10), obtained from the ten accessions of *G. monspessulana* (*Gm*1–10), are presented in Table 1. Studied extracts showed anti-proliferative activity against test cell lines at different levels, indicating that these *G. monspessulana* accessions might differentially produce cytotoxic compounds. The resulting IC_50_ values fell into the 26.3–500 µg/mL range. The ten extracts were not active against A549 (IC_50_ > 500 µg/mL) and practically inactive against fibroblasts (IC_50_ > 481 µg/mL), while PC-3 was the most susceptible cell line to the extracts (average IC_50_ = 98.3 ± 68.1 µg/mL), followed by SiHa (average IC_50_ = 148.8 ± 97.8 µg/mL). *Gm*10 accession was the most active extract (IC_50_ = 26.3 µg/mL) against the PC-3 cell line, having a good selectivity since it was inactive against fibroblasts. Indeed, the second accession group (i.e., *Gm*3,5,7,9,10) exhibited better activity (IC_50_ < 57.8 µg/mL) against PC-3 than the first group (*Gm*1,2,4,6,8; IC_50_ > 92.8 µg/mL). This trend was similar to SiHa and oppositely behaved against L929. These facts indicated that *G. monspessulana* seed-derived extracts exhibited selectivity to PC-3 and SiHA cancer cell lines. Additionally, their anti-proliferative activity was found to be differential depending on the seed origin, possibly due to the collecting environment (i.e., grassland versus woodland areas). In this regard, seeds retrieved from woodland-growing plants exhibited generally higher anti-proliferative activity than seeds from grassland-growing plants, conceivably due to the stimulation of metabolite production as an adaptive response to face the plausible biotic pressure of other competing organisms (e.g., plants, insects, microorganisms) [51]. Contrarily, plants in open spaces, having different pressure and good growth conditions, possibly promoted a more constitutive status in *G. monspessulana* plants [52].

### 2.2. Characterization Based on LC-ESI-MS Data of G. monspessulana Seed-Derived Extracts

**Figure 1:**
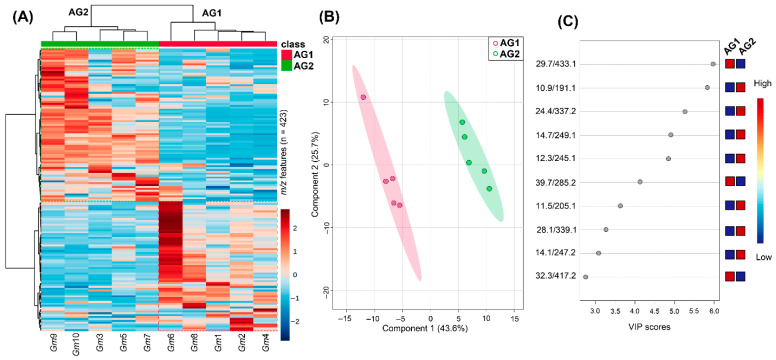
Chemical differentiation of the seed extracts from the ten *G. monspessulana*, using liquid chromatography coupled to mass spectrometry (LC-MS) profiles. AG1 = accession group 1; AG2 = accession group 2. (**A**) Heat-map-based chemical distribution of the *m/z* feature intensity detected in *G. monspessulana* seed extracts (*Gm*1–10). Columns organized each extract in the heat map. Each colored cell was associated with an autoscaled intensity of each detected *m/z* feature, depending on the color scale (dark red: high intensity; dark blue: low intensity). (**B**) Partial least square–discriminant analysis (PLS-DA)-derived score plot (component 1 (C1) vs. C2); accession grouping used as a categorical variable; variance explained = 69.3%. (**C**) PLS-DA-derived variable importance in the projection (VIP) plot; the ten most influential features were ranked (VIP scores > 2.5); retention time and mass/charge ratio (rt/*m/z*) pairs were used for the chemical identity of each top-ranked feature.

Reverse-phase liquid chromatography coupled to mass spectrometry using electrospray ionization (RP-LC-ESI-MS) was used to characterize the ten extracts from *G. monspessulana* accessions chemically. The recorded *m/z* features per extract were recovered from the LC-MS raw data and gathered into a feature intensity table (FIT). Thus, 423 features were compiled from the ten extracts, implying a high metabolite diversity. Extracts shared various metabolites (i.e., features) involving intensity variations. In contrast, other metabolites occurred in particular extracts. This observation was expanded by the global LC-MS-based metabolite distribution illustrated by a heat map. It was built under the classification based on accession groups (AGs) according to the seed origin, i.e., AG1 and AG2, and after scaling the feature intensity to unit variance (i.e., autoscaling) to define differential metabolites depending on the color scale (3 to −3), i.e., dark red (=3) related to high feature intensity and dark blue related (=−3) to low feature intensity (Figure 1A). 

According to the hierarchical clustering analysis (HCA) performed on the autoscaled metabolite data, the heat map evidenced that both AGs were discriminated by the presence and/or abundance of particular metabolites. Globally, accessions within an AG exhibited similar profiles, although some specific differences are also involved. In addition, the AG separation depending on feature amount was similar (i.e., the total number of features was almost equally divided between AG1 and AG2). *Gm*6 was the seed accession with the highest number of the most abundant metabolites, and *Gm*1 and *Gm*4 with the lowest number (Figure 1A). This fact suggested that the chemistry of *G. monspessulana* seeds is also influenced by the growth environment, as reported for other Fabaceae plants [53,54]. The most active extracts coincided with the AG2, and they exhibited more abundant metabolites than AG1 (which included the least active extracts), suggesting that AG2 extracts contain interesting compounds that are probably responsible for the observed anti-proliferative effect. 

An additional partial least square–discriminant analysis (PLS-DA) demonstrated such a chemical differentiation between the AGs, using the two first components (69.3% variance explained), involving a good separation as observed in the C1 vs. C2 score plot (Figure 1B). The PLS-DA-derived variable importance in the projection (VIP) demarcated such a contrast, the scores of which led to ranking the ten most influencing compounds in AG differentiation through a VIP plot (Figure 1C). These top-ranked features were registered by the retention time, and mass/charge ratio (rt/*m/z*) pairs since the annotation failed because the putative identification resulted in various isomers. 

Under this top ranking, nine features exhibited a high differential trend based on VIP scores (>3) as the criterion for selection. Therefore, two features were then related to the statistical separation of AG1 metabolite profiles, while seven features were related to AG2 distinction. Since the anti-proliferative activity was linked to the AG division, these seven top-ranked compounds that influenced the AG differentiation through chemical profiles can be considered active principal candidates. Consequently, the MS-based chemical and bioactivity datasets were statistically integrated to support this hypothesis and recognize the plausible anti-proliferative compounds.

### 2.3. Detection of Anti-Proliferative Candidates from G. monspessulana Accessions through the Integration of Chemical Fingerprint and Bioactivity Datasets

**Figure 2:**
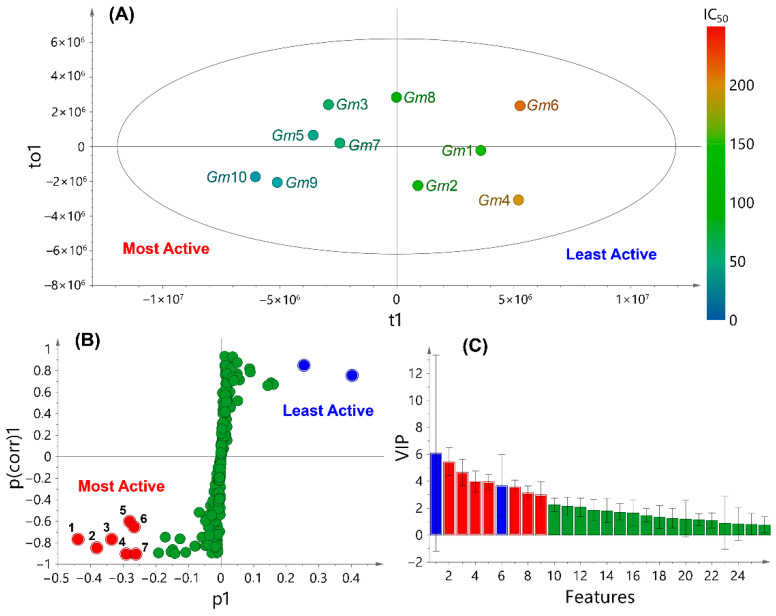
Integration of LC-MS-based fingerprints and anti-proliferative activity datasets of seed extracts from *G. monspessulana* accessions (*Gm*1–10) by single-Y orthogonal partial least squares (OPLS). IC_50_ values were used as a continuous Y variable, depicted as a color scale (red = 250 µg/mL; blue = 0 µg/mL). (**A**) Score plot. (**B**) S-plot. (**C**) VIP plot. Red dots in the S-plot, numbered as **1–7**, indicate the most influential variables.

To recognize such active metabolites produced by *G. monspessulana* seeds, the anti-proliferative activity (APA) and the LC-MS-based fingerprint (LMFP) datasets were integrated through multiple-covariate statistics. A single-*Y* orthogonal partial least squares (OPLS) regression was then used to associate such datasets. Due to the observed selectivity on the PC-3 cell line (Table 1), the respective IC_50_ values were specifically employed as the APA dataset and, consequently, the continuous *Y* variable. Thus, the resulting OPLS model, containing one predictive score (t1) and one orthogonal component (to1), differentiated the studied extracts based on APA (Y-data) and LMFP (X-data), exposing a well-fitted (R^2^_X_ = 0.883, R^2^_Y_ = 0.812) and predictable (Q^2^_Y_ = 0.721) model and explaining the variance by the APA (49.5% along t1) and LMFP (38.8% along to1). The OPLS-derived score plot (Figure 2A) revealed the respective discrimination mode of seed extracts from *G. monspessulana* accessions. Hence, the APA-influencing differentiation of metabolite profiles was visualized by the IC_50_ values using a color scale between red (250 µg/mL) and blue (0 µg/mL). Through this pattern, the most active extracts clustered on the left side, but contained clearly different profiles because of their high dispersion, while the least active extracts were located on the right side. This trend corroborated the previously observed fact that specific metabolites occurring in the most active extracts might be responsible for the observed APA. 

To facilitate the recognition of those compounds as bioactive candidates, the respective PLS-DA-derived loadings were scrutinized by employing an *S*-plot transformation (a p1 × p_(corr)_1 scatter plot forming an *S*-like contour) to categorize the relative importance of differential variables (i.e., metabolites). This *S*-plot (Figure 2B) displayed the covariance and the correlation structure between the X-data and t1 [55], using Pareto and centering scaling. Accordingly, the most important chemical differences among least active (p1 > 0) and most active (p1 < 0) *G. monspessulana* seed-derived extracts were exposed by the metabolites located distantly in the wings of the *S*-plot, showing a strong influence on the model with high reliability. Therefore, seven compounds (numbered as **1**–**7**, red dots) were categorized as the most influential variables (p_(corr)_1 > 0.4, p1 < –0.2) for the OPLS-based differentiation of the most active extracts. In contrast, the other two metabolites (blue dots) were highly related to the least active extracts. 

The resulting VIP plot (Figure 2C) corroborated such a relevant influence on the integrative discrimination as plausible bioactives (VIP scores > 3). These features showed *m/z* and rt values in the 191.1–339.1 and 10.9–39.7 ranges, respectively, including the rt/*m/z* pairs 191.1/10.9 (**1**), 337.2/24.4 (**2**), 249.1/14.7 (**3**), 245.1/12.3 (**4**), 205.1/11.5 (**5**), 339.1/28.1 (**6**), 247.2/14.1 (**7**). According to the combined LC and MS behavior, these metabolites were related to intermediate-polar and low-weight compounds. Compound **1** showed the most substantial model influence due to its high discriminating importance (p1 < –0.4). This compound was highly present in *Gm*9 and *Gm*10 extracts. Compounds **4** and **7** showed the best reliability on the basis of their differential p_(corr)_1 value, indicating that these compounds are frequent in various *G. monspessulana* extracts. Compounds **5** and **6** displayed the lowest model influence and reliability. Nevertheless, slight differences in these discriminating parameters were observed for the differential metabolites **1**–**7**. Therefore, they were considered within the pattern recognition as anti-proliferative candidates that probably participated in the measured APA against the PC-3 cell line by the studied extracts. This outcome indicated that APA/LMFP dataset integration could be successfully achieved for bioactive pinpointing by single-*Y* OPLS, since the covariance maximization of discriminating metabolites (independent variables) as a function of bioactivity (continuous or categorical dependent variable) is satisfactorily achieved by supervised statistical methods, e.g., OPLS or PLS, but not by unsupervised methods, e.g., principal component analysis (PCA) [56]. Thus, PCA was not employed as a first-line analysis. 

The main advantage of the dataset association based on metabolite profiling is using chemical fingerprints as the source of independent variables to be integrated with the bioactivity of mixtures of natural origin as a dependent variable [57]. Because single-*Y* OPLS uses a continuous variable, its convenience as a multiple-covariate dataset integration is higher than that of those using categorical variables since a considerable amount of relevant information can be lost [58,59]. In addition, this integration can also detect unstable metabolites, with this being the primary concern during an extract fractionation [48]. However, an intrinsic limitation is the detection of false positives due to the synergistic/antagonistic effects with other components within extracts [60]. Therefore, the targeted isolation of **1**–**7** was carefully performed to validate the observed correlative differentiation by assessing their anti-proliferative activity on cancer cell lines.

### 2.4. Isolation and Identification of OPLS-Recognized Anti-Proliferative Candidates

**Figure 3:**
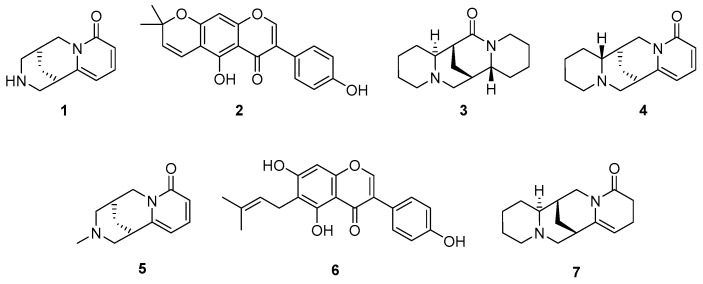
Structures of isolated compounds **1–7** after statistical pattern recognition from *G. monspessulana* seeds.

The fingerprint/bioactivity integration recognized metabolites **1**–**7** as the most discriminating metabolites for the most active APA-based extracts. Therefore, semipreparative HPLC separations were conducted to purify these compounds from the most active extracts (i.e., *Gm*9 and *Gm*10). Compounds **1**, **3**, and **4** were obtained from *Gm*10, whereas *Gm*9 extract afforded compounds **2**, **5**, **6**, and **7**. After isolation, the structures of compounds **1–7** were elucidated by diagnostic scrutiny of nuclear magnetic resonance (NMR) and MS data. Compounds were therefore identified, and their ^13^C NMR data were identical to those reported for the known metabolites (–)-cytisine (**1**) [61], alpinumisoflavone (**2**) [62], (+)-aphylline (**3**) [63], (–)-anagyrine (**4**) [64], (–)-*N*-methylcytisine (**5**) [65], wighteone (**6**) [66], and (+)-5,6-dehydrolupanine (**7**) [67]. The structures of **1**–**7** are presented in Figure 3.

The purified compounds were assessed against the four cell lines using the MTT viability assay to validate the OPLS-based bioactive recognition. Their resulting IC_50_ values are listed in Table 2. As expected, PC-3 was the most susceptible cell line when treated with compounds **1**–**7**, but the alkaloid **1** exhibited the most potent anti-proliferative effect (IC_50_ = 15.8 μM) on this cell line, but lower than positive control curcumin (IC_50_ = 9.5 µM). However, **1** showed a cytotoxic effect on A549 (IC_50_ = 42.5 μM) and fibroblasts (IC_50_ = 102.7 μM). The pyranoisoflavone **2** also displayed good activity against PC-3 (IC_50_ = 18.6 μM), but it was most active for SiHa (IC_50_ = 19.6 μM) and inactive against L929, while prenylated isoflavone **6** was the least active compound against PC-3 and also inactive for fibroblasts. Alkaloids **3**–**5** and **7** exhibited antiproliferative effect on PC-3 (21.3 μM > IC_50_ > 31.5 μM) but also fibroblasts (22.3 μM > IC_50_ > 91.3 μM). In addition, alkaloid **5** was the most active compound against the A549 cell line (IC_50_ = 36.3 μM). 

Cytisine-type alkaloids **1** and **5** revealed an interesting trend regarding the anti-proliferative activity. In this regard, a methyl group at N12 seemed to negatively affect the anti-proliferative activity against PC-3 and SiHa but had a positive effect against A549, which can be further investigated. In contrast, alkaloids **3**, **4**, and **7** and isoflavones **2** and **6** showed similar anti-proliferative profiles for each metabolite type, with an unclear trend. However, alkaloids were generally cytotoxic for fibroblasts (IC_50_ < 85.9 μM), while isoflavones were found to be inactive against L929 (IC_50_ > 400 μM).

Cytisine (**1**) is a well-known compound with several relevant biological properties, including cytotoxic and anti-proliferative activities [68], particularly against the HepG2 (human hepatocellular carcinoma) cell line, by inducing mitochondrial-mediated apoptosis [69]. Compound **1** was also reported to be cytotoxic through apoptosis induction against the A549 cell line, coinciding with our findings but involving better activity (IC_50_ = 26.83 µM) [70]. Other cancer cell lines, such as the FaDu, MCF-7, and MDA-MB cell lines, are not affected by **1** [71], which confirms its observed selectivity to lung and prostate cancer cell lines. Similarly, alpinumisoflavone (**2**) has several reports on various biological activities, including cytotoxicity against various cancer cell lines, such as human oral epidermoid (KB), murine leukemia (P-388), human leukemia (HL-60, K-562, MOLT-4), human lung (H2108, H1299, MRC-5), human renal (ccRCC 786-O, Caki1, SN12C), neuroblastoma (SH-SY5Y), human melanoma (A375, SK-MEL-1), human esophageal (Eca109, KYSE30), and colorectal (HCT-116, SW480), comprising IC_50_ < 70 µM [72]. In addition, compound **2** has also been reported to have activity against PC-3 with an IC_50_ < 30 µM [73], which coincided with our results. Anagyrine (**4**) showed cytotoxic activity against HL-60 cell line (IC_50_ = 18 µM) [74], while *N*-methylcytisine (**5**) can inhibit the proliferation of SMMC-7721 (hepatocellular carcinoma) cells [75], and wighteone (**6**) exhibited IC_50_ > 70 µM for HL-60, K-562, and MOLT-4 cell lines and even less active than alpinumisoflavone [76]. 

There are no records of aphylline (**3**) or 5,6-dehydrolupanine (**7**) possessing anti-proliferative activity. In addition, compounds **1** and **4**–**6** have no records on activity against PC-3, and none of the bioactives **1**–**7** have been evaluated against SiHa. Therefore, they were evaluated for the first time in the present study against these cell lines for the purposes of statistical integration. These findings confirmed that this association effectively identifies anti-proliferatives against these three cancer cell lines from *G. monspessulana* seed extracts, validating the candidates, supported by previous studies assessing the activity of some of these compounds against several cancer cell lines. However, there is a probability that other active compounds are missing due to the plausible antagonistic effects of diverse extract components, so deeper integrative analyses for detecting those missed bioactives are recommended, even those less active but having synergistic roles that could improve the activity [77]. Lastly, since the isolated compounds **1**–**7** were evaluated at different concentrations from their parent extracts, a direct comparison of anti-proliferative activity between individual compounds and extracts was not possible. Therefore, further studies would be necessary to disclose whether the isolated compounds **1**–**7** are the only anti-proliferative bioactives in the test *G. monspessulana* seed extracts.

**Figure 4:**
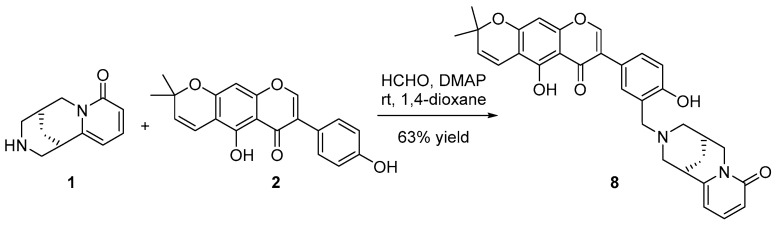
Synthesis of cytisine-linked isoflavonoid **8** by Betti reaction from cytisine (**1**) and alpinumisoflavone (**2**).

A recent study explored the inhibitory activity of prostate and colon cancer cell proliferation by cytisine-linked isoflavonoids (CLIFs) at C7 through a carbon chain (C2–C6) spacer showing inhibition > 60% at 10 µM [78]. We explored a similar strategy, considering that **1** and **2** were the most potent compounds against the PC-3 cell line (IC_50_ < 18.6 µM). Thus, we used a Betti-like reaction [79] to condense **1** and **2** into the CLIF **8** (a novel cytisine-alpinumisoflavone adduct) (Figure 4). This reaction involves a multi-component protocol to combine an aldehyde, a primary/secondary amine, and a phenol to produce *N*-substituted-2-aminomethylphenols. In this regard, the reaction proceeded at room temperature with the imine formation by nucleophilic addition of **1** (amine) to formaldehyde and, subsequently, **2** (nucleophilic phenol) was added to the resulting imine in the presence of 4-dimethylaminopyridine (DMAP) to afford the CLIF **8** (63% yield).

The synthetic compound **8** was also evaluated against the four cell lines. This compound showed an enhanced anti-proliferative profile, since the activity against the three cancer cell lines was better than that of precursors **1** and **2**, with PC-3 being the most susceptible cell line (IC_50_ = 10.1 µM). In addition, CLIF **8** exhibited better activity against SiHA and A549 (IC_50_ = 17.5 and 46.8 µM, respectively) but a lower anti-proliferative effect on fibroblasts (IC_50_ = 385 µM). The activity outcome for **8** comprised selectivity indexes (SI = IC_50_ normal cells/IC_50_ cancer cells) of 38.1, 22.0, and 8.2 for PC-3, SiHa, A549, and L929, respectively. Recently, a cytisine-linked pterocarpan (tonkinensine B) was synthesized from cytisine and (−)-maackiain [80]. This adduct exhibited ca. 2–3-fold better activity against two human breast cancer cell lines (i.e., MCF-7 and MDA-MB-231) and lower cytotoxicity against normal RAW 264.7 (mouse macrophages) and BV2 (mouse microglia) cell lines than respective precursors, coinciding with our findings. Tonkinensine B has cytotoxic activity by apoptosis induction, a relevant mechanism to be expected for anticancer compounds. In tonkinensine B, cytisine was linked to C4 at the pterocarpan’s A-ring through its OH at C3. Other previously studied CLIFs had the cytisine linked to C7 at the A-ring via an ether bridge. In contrast, CLIF **8** contained a phenolic OH at the B-ring, which favored the reaction with cytisine, since the OH at C5 does not have the proper chemical environment for the Betti reaction. Thus, derivatives of **8** can be further studied as a novel CLIF series to explore its potential against mainly prostate but also cervical and lung cancers.

## 3. Materials and Methods

### 3.1. Plant Material

Seeds of ten *G. monspessulana* (L.) L.A.S. Johnson were collected in Cundinamarca and Boyacá, Colombia, between July and September, 2014, from different growth conditions provided by surroundings, abiding by the Colombian ethical legislation. Collected seeds per accession were then subdivided into the two accession groups: (1) AG1 contained seeds of five accessions (*Gm*1,2,4,6,8) retrieved from plants growing in open spaces and grasslands forming dense scrub associations, and (2) AG2 contained those seeds of the other five accessions (*Gm*3,5,7,9,10), retrieved from plants growing in spaces near woodland and even under a tree canopy. Voucher specimens are kept at Colombian National Herbarium. The collected healthy seeds were transported to the laboratory for extract preparation. 

### 3.2. Extract Preparation

The healthy seeds (50 g) from the ten *G. monspessulana* accessions were separately air-dried, crushed, ground, and extracted with 96% ethanol at a constant shaking speed (120 rpm) using a Heidolph Rotamax 120 platform orbital shaker (Heidolph Instruments GmbH & Co.KG, Schwabach, Germany). The extraction lasted one week, with daily filtration-mediated removal of the extract-containing solvent and replaced by fresh 96% ethanol. The filtered solution was concentrated by distillation under reduced pressure at 40 °C using an IKA RV 10 Control rotary evaporator (IKA^®^ RV 10, IKA^®^ Werke GmbH & Co. KG, Staufen, Germany) to afford the raw extracts, which were compiled per accession after each daily extraction. The resulting raw extracts per accession were dried and stored at −20 °C until subsequent biological and chemical analyses.

### 3.3. In Vitro Cell Viability Assay

Human prostatic adenocarcinoma (PC-3, ATCC CRL-7934), human lung adenocarcinoma (A549, ATCC CCL-185), and human cervical carcinoma (SiHa, ATCC HTB-35) cancer cell lines and normal mouse fibroblasts (L929, ATCC CRL-6364) were maintained in a humidified atmosphere with 5% CO_2_ at 37 °C, and grown as a monolayer culture in Dulbecco’s Modified Eagle Medium (DMEM) medium with 10% (*v*/*v*) fetal bovine serum (FBS), 1% (*v*/*v*) penicillin, and 1% (*v*/*v*) streptomycin. The anti-proliferative effects of *G. monspessulana* extracts and isolated compounds were measured according to a reported method [81]. Cell suspension (100 µL, 5 × 10^3^ cells/well) was inoculated in 96-well plates and cultured for 24 h. After that, the culture medium was replaced with a serum-free medium (100 µL) containing different concentrations of treatments (0.8–500 µg/mL for extracts and 0.16–100 µg/mL for pure compounds). Extracts and compounds were measured in triplicate. A PBS-containing free medium was used as a blank, 1% (*w*/*v*) bovine serum albumin-amended medium as negative control (100% survival), and curcumin (0.16–100 µg/mL) was used as the positive control. After 48 h of incubation, the cell viability was assessed by adding MTT (10 µL, 5 mg/mL) to each well, and the plates were subsequently incubated under 5% CO_2_ at 37 °C for 3 h. Formazan crystals were dissolved with 100 µL of DMSO. Absorbance was measured at 570 nm using a Varioskan LUX 96-well plate reader (Thermo Fisher Scientific, Waltham, MA, USA). The anti-proliferative effects were expressed as half-maximal inhibitory concentration (IC_50_) in µg/mL (extracts) and µM (compounds). The IC_50_ values were calculated from the dose-response curves through non-linear regression using GraphPad 5.0 (GraphPad Software, San Diego, CA, USA).

### 3.4. High-Performance Liquid Chromatography Coupled to Mass Spectrometry

Metabolite profiles of test extracts were recorded on a Shimadzu Prominence (Shimadzu Corporation, Kyoto, Japan) equipped with two binary pumps, an autoinjector, a photodiode array (PDA) detector, and an LCMS2020 mass spectrometry detector with a single quadrupole analyzer and electrospray ionization (ESI). Each seed extract was dissolved in absolute ethanol (5 mg/mL) and injected (20 µL) into the HPLC system. The separation system consisted of a Synergi C18 column (Phenomenex, Torrance, CA, USA) (4.6 mm × 150 mm, 4 μm), and a combination of solvent A (1% formic acid in Mili-Q H_2_O) and solvent B (1% formic acid in acetonitrile (ACN)). A gradient elution method at 0.7 mL/min was used as follows: 0–2 min 5% B, 2–20 min 5% to 40% B, 20–27 min 40% B, 27–42 min 40% to 90% B, 42–46 min 90%, and 46–50 min 90% to 5% B. The monitoring wavelength was 270 nm. Mass spectra were simultaneously acquired using electrospray ionization in the positive ion mode (scan 100–2000 *m/z*). The MS parameters involved a voltage detector (1.5 kV), curved desolvation line (CDL) (250 °C), heat block temperature (400 °C), and a nebulization gas flow (1.5 L/min).

### 3.5. LC-MS-Derived Fingerprint Processing

MS data obtained from LC-ESI-MS were processed in MZmine 2.17, comprising peak detection, baseline correction, and deconvolution [82], using the following parameters: mass detection (centroid; MS^1^ noise level = 1 × 10^5^), automated data analysis pipeline (ADAP) chromatogram builder (minimum group size in # scan: 5; group intensity threshold: 1 × 10^7^; minimum highest intensity: 1 × 10^7^; *m/z* tolerance: 0.5), chromatogram deconvolution (minimum peak height: 5 × 10^5^; peak duration: 0 to 2 min; baseline level: 1 × 10^5^) isotope grouping (*m/z* tolerance: 0.5; retention time (RT) tolerance: 0.2; max charge: 2), joining aligner (*m/z* tolerance: 0.5; weight for *m/z*: 70; RT tolerance: 0.2; weight for RT time: 30), peak filtering (minimum peak in row: 5; *m/z* range: 75–2000; reset peak number ID: true), gap filling (intensity tolerance: 10%; *m/z* tolerance: 0.5; RT tolerance: 0.2).

### 3.6. Multiple-Covariate Integration of Chemical Fingerprint and Bioactivity Datasets

The processed data was exported in csv format to build the feature intensity table (FIT), i.e., (10 samples × 423 features), and the data were autoscaled (unit variance scaling) to perform suitable comparisons. A heat map was built to intuitively visualize the autoscaled feature distribution using MetaboAnalyst 5.0 (McGill University, Quebec, Canada) [83]. The pre-treated FIT was then joined with the respective anti-proliferative activity data (i.e., APA as a continuous variable) to assemble the integrated dataset. The resulting matrix was then imported into the SIMCA software (v 14.0) (Umetrics, Umeå, Sweden) to build the respective models by single-*Y* orthogonal partial least squares (OPLS). The obtained results were visualized using scores and *S* plots.

### 3.7. Purification and Identification of Anti-Proliferatives **1**–**7** by Semipreparative HPLC

*Gm*9 and *Gm*10 extracts (500 mg) were independently treated with solid-phase extraction (SPE) Strata^®^ C18-U cartridges (55 µm, 70 Å, 500 mg, 6 mL) (Phenomenex, Torrance, CA, USA). These SPE cartridges were previously conditioned with methanol (6 mL) and then water (6 mL). After loading the extracts, the cartridges were washed with water (5 mL). The adsorbed components were eluted with methanol (5 mL). Collected eluates containing depurated extracts were used for semi-preparative HPLC-mediated isolation after geometrical transfer from optimized analytical conditions to maintain resolution and profile. Thus, the target compounds **1**–**7** were isolated using a UFLC Prominence system (Shimadzu, Columbia, MD, USA), operated in a semipreparative mode, consisting of a pump (LC-20AD), a column oven (CTO-20AC), a UV/Vis detector (SPD-20AV), an autosampler (SIL-10AP), a fraction collector (FRC-10A) and equipped with a reversed-phase Phenomenex Luna C_18_ column (250 × 10 mm, 5 μm) (Phenomenex, Torrance, CA, USA) at 20 °C. Ten consecutive injections of SPE-depurated extract (500 μL per injection, 80 mg/mL in MeOH) were separated at a flow rate of 3 mL/min using solvents A (1% formic acid in H_2_O) and B (1% formic acid in ACN) using an isocratic elution method. Targeted peaks, according to the OPLS-based recognition, were collected at retention time ranges of 10.8–11.0 min (5.5 mg, **1**), 11.3–11.7 min (6.6 mg, **5**), 12.1–12.5 min (4.7 mg, **4**), 13.9–14.2 min (3.8 mg, **7**), 14.6–14.9 min (10.5 mg, **3**), 24.3–24.5 min (4.3 mg, **2**), and 28.0–28.2 min (5.7 mg, **4**), to afford pure compounds. *Gm*10 extract yielded compounds **1**, **3**, and **4**, and *Gm*9 extract afforded compounds **2**, **5**, **6**, and **7**. Structures of isolated compounds were elucidated by ^1^H and ^13^C NMR, through the attached proton test (APT), on an Avance 400 spectrometer (Bruker, Billerica, MA, USA) using CDCl_3_ as solvent (400 Mz ^1^H; 100 MHz ^13^C). All shifts are given in δ (ppm) using tetramethylsilane (TMS) as the internal standard. Coupling constants (*J*) are given in Hz. APT ^13^C NMR data and optical rotation of isolated compounds were identical to that reported for (–)-cytisine (**1**) [61], alpinumisoflavone (**2**) [62], (+)-aphylline (**3**) [63], (–)-anagyrine (**4**) [64], (–)-*N*-methylcytisine (**5**) [65], wighteone (**6**) [66], and (+)-5,6-dehydrolupanine (**7**) [67].

### 3.8. Synthesis of Cytisine-Linked Isoflavonoid 8

Into a round-bottom flask, (–)-cytisine (0.02 mmol), alpinumisoflavone (0.01 mmol), and 1,4-dioxane (2 mL) were added and mixed at room temperature to afford a solution. Subsequently, formaldehyde (0.02 mmol) and DMAP (1% mmol) were also added. The reaction mixture was stirred at room temperature until completion, determined by thin-layer chromatography using silica gel plates and a chloroform/methanol (98:2) solvent mixture as a mobile phase. Subsequently, the solvent was removed under reduced pressure to afford the crude reaction product. Compound **8** was purified by column chromatography on silica gel (chloroform/methanol 98:2). NMR and MS analyses confirmed the structure of the target compound **8**. HPLC analysis (Synergi C18 column (4.6 × 150 mm, 4 μm), flow rate at 0.7 mL/min, 1% formic in acetonitrile gradient, and photodiode array (PDA) detector at 270 nm) led to verify the purity of **8** (>99%).

(–)-3′-(*N*-cytisine)methylalpinumisoflavone (**8**): (63% yield). Amorphous solid. [α]_D_^20^ = –25.6 (CHCl_3_, c = 0.005). ^1^H NMR (CDCl_3_, 400 MHz) δ_H_ 8.13 (1H, s, H-2), 7.53 (1H, d, *J* = 2.4 Hz, H-2′), 7.41 (1H, dd, *J* = 9.0, 6.7 Hz, H-4‴), 7.31 (1H, dd, *J* = 8.8, 2.4 Hz, H-6′), 6.95 (1H, d, *J* = 8.8 Hz, H-5′), 6.70 (1H, d, *J* = 10.3 Hz, H-1″), 6.48 (1H, dd, *J* = 9.0, 1.3 Hz, H-3‴), 6.37 (1H, s, H-8), 6.22 (1H, dd, *J* = 6.7, 1.3 Hz, H-5‴), 5.73 (1H, d, *J* = 10.3 Hz, H-2″), 4.11 (1H, d, *J* = 15.5 Hz, H-10‴a), 3.89 (2H, s, H-14‴), 3.87 (1H, dd, *J* = 15.5, 6.9 Hz, H-10‴b), 3.11–3.15 (1H, m, H-7‴), 3.06 (1H, dd, *J* = 12.5, 2.6 Hz, H-13‴b), 2.99 (1H, dd, *J* = 12.0, 1.2 Hz, H-11‴b), 2.39–2.47 (3H, m, H-9‴), 1.90–195 (2H, m, H-8‴), 1.47 (6H, s, H-4″, H-5″). APT ^13^C NMR (CDCl_3_, 100 MHz) δ_C_ 181 (C-4), 165.5 (C-2‴), 159.7 (C-5), 158.2 (C-4′), 157.8 (C-9), 156.8 (C-7), 154.4 (C-2), 151.9 (C-6‴), 141.3 (C-4‴), 131.5 (C-1′), 129.7 (C-2″), 129.3 (C-6′), 127.1 (C-2′), 123.7 (C-3), 117.9 (C-3‴), 116.8 (C-5′), 114.9 (C-1″), 114.7 (C-3′), 108.5 (C-5‴), 106.3 (C-10), 105.4 (C-6), 95.5 (C-8), 78.1 (C-3″), 62.9 (C-13‴), 61.8 (C-11‴), 55.3 (C-14‴), 49.9 (C-10‴), 36.1 (C-7‴), 28.8 (C-9‴), 27.5 (C-4″), 27.5 (C-5″), 27.4 (C-8‴). HRESIMS [M+H]^+^ *m/z* 539.2169 (calcd. for C_32_H_31_N_2_O_6_, 539.2182).

## 4. Conclusions

Seed extracts from ten *G. monspessulana* accessions exhibited anti-proliferative activity against three cancer cell lines (i.e., PC-3, SiHa, and A549) at different levels, demonstrating selectivity to prostatic adenocarcinoma (PC-3) and cervical carcinoma (SiHa) cell lines and less toxicity on fibroblasts. To the best of our knowledge, the present study constitutes the first attempt to evaluate the inhibitory capacity of the studied *G. monspessulana*-derived extracts against cancer cell lines, affording selectivity to PC-3 cell lines. In addition, anti-proliferative activity revealed a differential pattern depending on the seed origin, probably due to the growing area of parent plants. Thus, seeds retrieved from woodland-growing plants were significantly more active against PC-3 than seeds from grassland-growing plants. This trend was rationalized through a specialized metabolite-mediated adaptive response to face plausible biotic pressures by other woodland-competing organisms, while plants in open spaces promoted a more constitutive status. This information was used as a bioactivity input for the indirect detection of plausible anti-proliferative candidates through metabolite profiling by relating the fingerprints and the PC-3-oriented anti-proliferative activity datasets, leading to the recognition of seven hits, such as (–)-cytisine (**1**), alpinumisoflavone (**2**), (+)-aphylline (**3**), (–)-anagyrine (**4**), (–)-*N*-methylcytisine (**5**), wighteone (**6**), and (+)-5,6-dehydrolupanine (**7**), as the active principle candidates. The isolation of these hits from active seed extracts and their anti-proliferative activity assessment demonstrated the effectiveness of this indirect approach based on LC-MS-based profiles to find bioactives from *G. monspessulana* extracts. Further explorations on *Genista* active extracts or individual compounds comprising quinolizidines and prenylated isoflavonoid moieties should be conducted to expand their potential as protective agents against cancer. Indeed, the most active compounds (**1** and **2**) were transformed into a more active compound, cytisine-linked isoflavonoid **8**, which displayed better activity against PC-3 with a good selectivity index (>30). Thus, **8**-related CLIFs might be considered in future studies to define their potential against lung, cervical, and prostate cancer.

## Figures and Tables

**Table 1:** Anti-proliferative activity against cancer cell lines and fibroblasts of seed extracts from ten *G. monspessulana* accessions.

	PC-3 ^b^		SiHa ^b^		A549 ^b^		L929 ^c^	
Samples ^a^	IC_50_^c^	CI ^d^	IC_50_^c^	CI ^d^	IC_50_^c^	CI ^d^	IC_50_^c^	CI ^d^
*Gm*1	151	141–165	136	124–151	>500	-	>500	-
*Gm*2	119	107–131	271	246–290	>500	-	313	291–328
*Gm*3	57.8	52.6–63.0	75.1	69.8–83.4	>500	-	>500	-
*Gm*4	196	186–210	216	194–233	>500	-	389	369–416
*Gm*5	42.8	39.4–47.1	61.4	57.1–68.2	>500	-	>500	-
*Gm*6	211	190–228	285	271–299	>500	-	226	206–251
*Gm*7	55.6	50.6–61.2	77.8	70.8–84.0	>500	-	>500	-
*Gm*8	92.8	87.2–99.3	258	245–276	>500	-	390	362–424
*Gm*9	31.6	29.7–33.5	48.4	43.6–53.2	>500	-	>500	-
*Gm*10	26.3	24.2–29.2	60.0	56.4–63.6	>500	-	>500	-

^a^ Seed-derived extracts from *G. monspessulana* accessions (*Gm*1–10); ^b^ test cancer cell lines: PC-3 (prostate adenocarcinoma), SiHa (cervical carcinoma), A549 (lung carcinoma); ^c^ normal cell line: L929 (fibroblasts); ^c^ Values expressed as half-maximal inhibitory concentration (IC_50_) in µg/mL; ^d^ CI = confidence interval (95% confidence) of the IC_50_ after non-linear regression.

**Table 2:** Anti-proliferative activity against cancer cell lines and fibroblasts of compounds **1**–**7** isolated from *G. monspessulana* seeds.

	PC-3 ^b^		SiHa ^b^		A549 ^b^		L929 ^c^	
Samples ^a^	IC_50_^c^	CI ^d^	IC_50_^c^	CI ^d^	IC_50_^c^	CI ^d^	IC_50_^c^	CI ^d^
**1**	15.8	14.7–16.6	357	341–388	42.5	39.5–45.5	102.7	97.6–107
**2**	18.6	17.9–20.1	19.6	17.6–20.6	>303	-	>297	-
**3**	21.3	19.2–23.2	>403	-	>403	-	22.3	19.8–25
**4**	33.5	31.8–35.8	>409	-	>409	-	36.5	33.6–38.7
**5**	28.4	26.4–31.5	>490	-	36.3	34.5–40.7	91.3	85.8–95
**6**	34.3	32.2–35.7	>296	-	>296	-	>296	-
**7**	31.5	29.6–33.1	>406	-	>406	-	85.9	79–95.3
**curcumin**	9.5	8.8–10.6	6.5	6.2–6.9	8.4	7.7–8.7	104.8	93.3–116

^a^ Isolated compounds **1–7** from *G. monspessulana* seeds (Figure 3); curcumin as positive control; ^b^ test cancer cell lines: PC-3 (prostate adenocarcinoma), SiHa (cervical carcinoma), A549 (lung carcinoma); ^c^ normal cell line: L929 (fibroblasts); ^c^ values expressed as half-maximal inhibitory concentration (IC_50_) in µM; ^d^ CI = confidence interval (95% confidence) of the IC_50_ after non-linear regression.

## Data Availability

The data that support the findings of this study are available from the corresponding author upon reasonable request.

## Sample Availability

Samples of the compounds **1–8** are available from the authors upon reasonable request.
